# Can Clinical Measures of Postoperative Binocular Function Predict the Long-Term Stability of Postoperative Alignment in Intermittent Exotropia?

**DOI:** 10.1155/2020/7392165

**Published:** 2020-07-21

**Authors:** Yidong Wu, Meiping Xu, Junxiao Zhang, Jinjing Zhou, Minghui Wan, Zhiyue Dai, Tingting Peng, Seung Hyun Min, Fang Hou, Jiawei Zhou, Xinping Yu

**Affiliations:** ^1^The Eye Hospital, School of Ophthalmology and Optometry, Wenzhou Medical University, Wenzhou, Zhejiang, China; ^2^McGill Vision Research, Department of Ophthalmology and Visual Sciences, McGill University, Montreal, QC, Canada

## Abstract

**Purpose:**

To evaluate whether clinical measures of postoperative binocular functions could predict the long-term stability of postoperative ocular alignment in children with intermittent exotropia.

**Methods:**

A retrospective study was performed in thirty-nine children (median: 7 years) who have been surgically treated from intermittent exotropia without overcorrection (less than 10 prism diopters [pd] of exodeviation at 1 month postoperatively). Angles of deviation and binocular functions were measured preoperatively and at 1 month, 6 months, and the final follow-up visit (≥24 months) postoperatively. We examined the relationships between postoperative drift (change of ocular alignment) and binocular functions (sensory fusion, fusional convergence amplitude, and stereoacuity).

**Results:**

The surgical success rate (esophoria/tropia ≤5 pd to exophoria/tropia ≤10 pd) dropped to 76.9% at 6 months after surgery and to 53.8% at individuals' last visit (mean: 37 months). The mean exodrift was 7.7 ± 9.2 pd from the postoperative month 1 to the final visit (*p* < 0.001) on distance fixation. Distance stereoacuity, central fusion, and fusional convergence amplitude significantly improved following surgery (*p* < 0.05). However, no significant correlation was found between their binocular functions measured at the beginning of each follow-up period and the postoperative drift (all *p* > 0.13).

**Conclusion:**

Our findings suggest that the clinical measures of sensory fusion, fusional convergence amplitude, and stereoacuity cannot serve as a robust predictor for the long-term stability of postoperative ocular alignment in patients who underwent successful surgery without overcorrection at 1 month postoperatively.

## 1. Introduction

Intermittent exotropia (IXT), a disorder that causes either of the eyes to drift outward spontaneously [1, 2], is the most common form of childhood exotropia [3]. It affects approximately 1% of children in the United States [3] and up to 3.5% in Asia [4]. Fusional compensatory mechanisms have been suggested to maintain eye alignment in this type of strabismus [1, 5, 6], allowing the development of binocular function. A common approach for clinicians to treat the patients with a poor control of exodeviation is strabismus surgery [1, 2, 7]. However, whether strabismus surgery benefits the patients longitudinally is nebulous because the recurrence rate of IXT after surgery has been shown to be high [8–11].

Previously, clinicians have investigated many factors that might be linked with IXT recurrence, such as the age of onset, refractive errors, visual acuity, preoperative angle of deviation, oblique dysfunction, lateral incomitance, and early overcorrection [2, 9, 10, 12]. However, none of the factors seems to influence the long-term outcome of the surgery [13].

Binocular functions (e.g., sensory fusion [14], stereoacuity [15], and fusional convergence [6]) are important for the management of IXT [1, 2]. Clinicians have assessed them in patients to determine the severity of IXT and, therefore, optimize treatment [2]. Moreover, accumulating evidence shows that patients with better binocular functions (sensory fusion, stereoacuity) preoperatively could achieve superior sensory outcomes postoperatively [14, 16, 17]. Additionally, one of the studies [14] indicates a trend toward inferior motor outcomes (i.e., recurrence) in patients with central suppression preoperatively. These findings suggest the predictive value of binocular functions. Given these previous observations, we asked a specific question in this study: can better postoperative binocular functions result in more stable ocular alignment following surgery in patients with IXT? We hypothesized that patients with better binocular functions following surgery could show a more stable binocular alignment. Indeed, clinicians have long assumed that the defect of fusional mechanisms might be responsible for the etiology of IXT [1, 18] and classified IXT as a cortical disorder of vergence [19]. We speculated that patients who showed poor binocular functions after alignment might have lost sensory neurons that normally support binocular function. Moreover, these neurons help maintain alignment by triggering vergence reflexes [20]. To answer the question, we designed a retrospective study to explore the relationship between clinical measures of postoperative binocular function and the stability of postoperative ocular alignment.

## 2. Methods

This retrospective study was approved by the ethical committee of the Eye Hospital of Wenzhou Medical University and conformed to the Declaration of Helsinki.

### 2.1. Patients

We reviewed the medical records of children below 16 years old who underwent strabismus surgery for IXT between January 2011 and December 2014 retrospectively. All surgeries were performed by one of the authors (XY). Clinical studies [9, 11, 12, 21] have suggested that early overcorrection may lead to binocular alignment within a certain follow-up period (e.g., 24 months). Therefore, we included patients whose eyes had been successfully corrected without overcorrection (defined as 0–10 prism diopters [pd] exodeviation for both distance and near fixation) at 1 month postoperatively. The patients were followed up at 6 months postoperatively and with a minimum follow-up of 24 months. We excluded patients who either had an A-V pattern, vertical deviation, oblique dysfunction, and/or dissociated vertical deviation (DVD). We also excluded patients with a history of prior strabismus surgery or had reoperation during follow-up period, as well as those with amblyopia (≥ 2 lines interocular difference by Snellen's vision chart), anisometropia (a spherical or cylindrical difference ≥ 2 diopters), neurologic abnormality, and/or developmental delay.

### 2.2. Data Collection

The preoperative and operative characteristics, including gender, age at surgery, best-corrected visual acuity, and cycloplegic refraction, as well as the surgical method, were obtained from the patients' medical records. The following parameters were collected preoperatively, 1 month postoperatively, 6 months postoperatively, and the individual's final follow-up: angle of deviation, sensory fusion status, fusional convergence amplitude, and stereoacuity. Angle of deviation in pd was measured using the prism and alternate cover test (PACT) at distance (6 m) and near (1/3 m) under spectacle correction. Sensory fusion was evaluated with Worth's 4-dot test at distance and near. Near stereoacuity was assessed using TNO test (Laméris Ootech B.V., Nieuwegein, the Netherlands), which ranges from 15 to 480 seconds of arc (arcsec). Distance stereoacuity was assessed via an Optec 3500 instrument (Stereo Optical Co., Chicago, IL, USA), ranging from 20 to 400 arcsec. Stereoacuity was recorded as “nil” if patients could not pass the largest disparity. Fusional convergence was detected via a synoptophore with fusion slides [22]. Patients were first instructed to move the tube to create a composite image (i.e., simultaneous perception). The fusional convergence was then tested by locking the columns at this corrected angle and converging the tubes until either “control” (a component in fusion slide) disappeared or the image split into two segments (i.e., break point). The amplitude of fusional convergence was calculated as the break point minus the point of simultaneous perception. The measurements in synoptophore were recorded in degrees and they were transferred into prism diopters. Patient's PACT was performed at the end of each visit to avoid potential effect of disrupting fusion; an additional 1-hour occlusion test was employed if needed to distinguish pseudodivergence excess type of IXT preoperatively [2].

### 2.3. Analysis

Surgical success was defined as the distant alignment in primary position, of esophoria/tropia ≤5 pd to exophoria/tropia ≤10 pd [9]. The postoperative drift was defined as the change of ocular alignment from month 1 to month 6, month 1 to final follow-up, and month 6 to final follow-up, respectively. The Friedman test was used to compare the angle of deviation, stereoacuity, and fusional convergence amplitude (pre-, 1 month, 6 months, and final follow-up). We performed post hoc analyses using the Wilcoxon signed-rank test. Sensory fusion status at each time point (pre-, 1 month, 6 months, and final follow-up), including post hoc analysis, was evaluated using a Chi-square test or a Fisher exact test. Patients were divided into two subgroups based on their sensory fusion status [14] at each time point (pre-, 1 month, and 6 months): present (4 dots) and absent (3 or 2 dots). We also classified patients into another set of two subgroups based on their stereoacuity [17] at each time point (pre-, 1 month, and 6 months): high-grade (≤60 arcsec) and moderate-low-grade (>60 arcsec). The Wilcoxon rank-sum test was used to compare the postoperative drift between subgroups. Besides, we evaluated the relationships between patients' characteristics, sensory fusion, fusional convergence amplitude, stereoacuity, and postoperative drift using the Spearman correlation coefficient. Stereoacuity was transformed to log units when we analyzed it as a continuous outcome. Patients with nil stereoacuity were assigned to the next highest 0.3 log increment level (i.e., 960 arcsec for TNO and 800 arcsec for Optec 3500) [23]. All statistical analyses were performed using SPSS version 22.0 (SPSS; Chicago, IL, USA). A *p* value of 0.05 was established as significant. An *α* value was adjusted by the Bonferroni correction during multiple comparisons.

## 3. Results

A total of thirty-nine cases were included. The median age of the patients at surgery was 7 (range, 3–16) years old, and the mean follow-up was 37.2 ± 7.9 (mean ± standard deviation [sd]; range, 24–52) months. Mean preoperative exodeviation was 34.4 ± 10.1 (95% CI, 31.1–37.7) pd at distance and 38.8 ± 9.0 (95% CI, 35.9–41.7) pd at near. A summary of patients' demographics and clinical characteristics is provided in Table 1.

### 3.1. Surgical Success Rate and Postoperative Drift

The success rate at 6 months postoperatively was 76.9% and at final visit was 53.8%.  Table 2 shows the success rate for different surgical types.

There was a statistically significant difference in the angle of deviation (*p* < 0.001) measured on distance fixation between each time point (pre-, 1 month, 6 months, and final follow-up). The mean exodrift was 3.4 ± 6.5 (95% CI, 1.3–5.5) pd from postoperative month 1 to postoperative month 6 (*p* = 0.004, adjusted *α* = 0.0083) and 7.7 ± 9.2 (95% CI, 4.7–10.7) pd from postoperative month 1 to final follow-up (*p* < 0.001; adjusted *α* = 0.0083).

Similarly, there was also a statistically significant difference in the angle of deviation (*p* < 0.001) measured on near fixation between each time visit point (pre-, 1 month, 6 months, and final follow-up). The mean exodrift was 3.2 ± 5.6 (95% CI, 1.4–5.0) from postoperative month 1 to postoperative month 6 (*p* = 0.001; adjusted *α* = 0.0083) and 7.9 ± 9.4 (95% CI, 4.8–10.9) pd from postoperative month 1 to final follow-up (*p* < 0.001; adjusted *α* = 0.0083).

### 3.2. Binocular Functions after Surgery

#### 3.2.1. Sensory Fusion

Preoperatively, 13 (33.3%) of 39 patients exhibited normal central fusion (4 dots) at distance. One month postoperatively, 30 (76.9%) of 39 patients exhibited normal central fusion (*p* < 0.001; adjusted *α* = 0.0083). The results remained stable at 6 months postoperatively (31 [79.5%]) and final follow-up visit (31 [79.5%]), which was not significantly different compared with that measured at 1 month postoperatively (*p* = 0.784 and 0.784, respectively; adjusted *α* = 0.0083). Preoperatively, 29 (74.4%) of 39 patients exhibited normal peripheral fusion (4 dots) at near. At one month postoperatively, 35 (89.7%) of 39 patients exhibited a normal peripheral fusion (*p* = 0.138; adjusted *α* = 0.0083). This rate slightly increased to 37 (94.9%) of 39 at 6 months postoperatively and remained constant at the final follow-up visit (37 [94.9%]). The peripheral fusion at 6 months postoperatively and the final follow-up visit was significantly different from that measured at 1 month postoperatively (*p* = 0.025; adjusted *α* = 0.0083).

Fifteen (38.5%) patients showed peripheral fusion and central suppression (i.e., 2 or 3 dots at distance and 4 dots at near) before surgery. The number of cases was 5 (12.8%) at 1 month postoperatively, 4 (10.3%) at 6 months postoperatively, and 6 (15.4%) at the final visit.

#### 3.2.2. Stereoacuity

Patients' median distance stereoacuity improved from nil (range, 30 arcsec to nil) before surgery to 70 arcsec (range, 20 arcsec to nil) at 1 month postoperatively (*p* < 0.001). Patients' distance stereoacuity remained stable at 6 months postoperatively and final follow-up compared with their performance at 1 month postoperatively (*p* = 0.904 and 0.928, respectively; adjusted *α* = 0.0083).

Patients' median near stereoacuity was 60 arcsec (range, 60 arcsec to nil) before surgery, in which 3 (7.7%) of 39 patients had unmeasurable (nil) stereoacuity. Their near stereoacuity did not significantly change following surgery at any follow-up compared with preoperative value (*p* = 0.401). Twenty-seven of 39 (69.2%) patients already had good near stereopsis (≤ 60 arcsec) before surgery and might have limited potential to further improve their near stereoacuity after surgery; we thus conducted an additional analysis on patients who had a moderate-low-grade (> 60 arcsec) stereoacuity before surgery (12 of 39, 30.8%). A Friedman test showed that these patients' near stereoacuity was also not significantly changed after surgery (*p* = 0.118).

#### 3.2.3. Fusional Convergence Amplitude

The mean value of the fusional convergence amplitude improved from the preoperative value of 6.6 ± 11.3 (range, 0–57.7) pd to 16.6 ± 13.0 (range, 0–64.9) pd at 1 month postoperatively (*p* < 0.001). The improvement remained stable at 6 months (22.6 ± 24.3, range, 0–83.9 pd) postoperatively and the final visit (25.0 ± 26.6, range, 0–83.9 pd) compared with their performance at 1 month postoperatively (*p* = 0.451 and 0.179, respectively; adjusted *α* = 0.0083).

### 3.3. The Relationship between Postoperative Binocular Functions and Postoperative Drift

#### 3.3.1. Postoperative Drift between Subgroups

For a given follow-up period (i.e., from postoperativemonth 1 to month 6; from postoperative month 1 to the final follow-up and from postoperative month 6 to the final follow-up), we calculated individual's exodrift in this duration. We then divided the patients into 2 subgroups based on their binocular functions (i.e., fusion and stereoacuity, see Methods for detail), which were measured at the beginning of this follow-up period. Those patients who reported seeing 5 dots on the Worth 4-dot test could have fusion capabilities. Therefore, we excluded them from the analysis (4 cases at 1 month postoperatively and 2 cases at 6 months postoperatively on near measurement; 4 cases at 1 month postoperatively and 4 cases at 6 months postoperatively on distance measurement). We conducted two-side Wilcoxon rank-sum tests to evaluate the difference of the postoperative drift between the 2 subgroups. We found that patients' postoperative drifts were not significantly different between those who had fusion (4 dots) and those had no fusion (3 or 2 dots). Patients' postoperative drifts were also not significantly different between those who had a good stereo (≤ 60 arcsec) and those who had a poor stereo (> 60 arcsec). These were true at any follow-up sessions and were true for both the near measures and distance measures. Detailed results are provided in Table 3.

#### 3.3.2. Associations between Stereoacuity, Sensory Fusion Status, Fusional Convergence Amplitude, and Postoperative Drift

**Figure 1:**
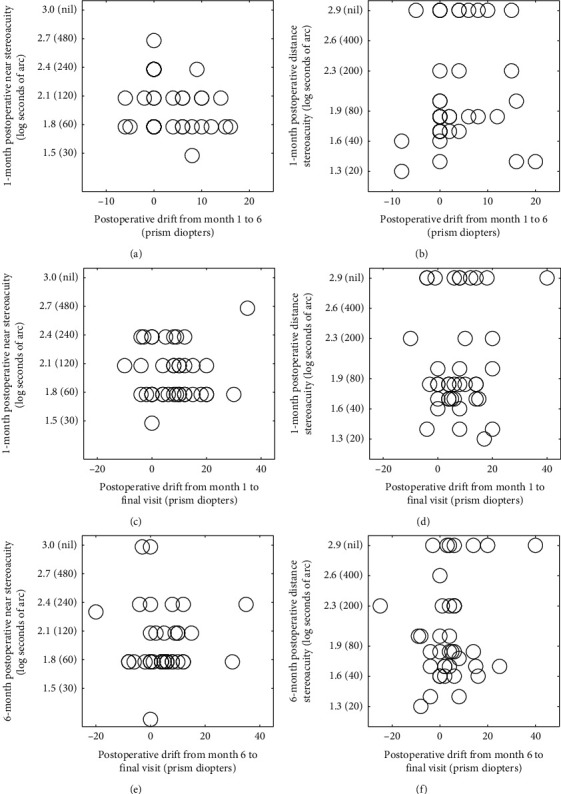
Relationships between postoperative stereoacuity and postoperative drift at the same distance. Each panel contains data from 39 patients. Stereoacuity was measured at the beginning of each follow-up period. A positive value indicates an exodrift and a negative value indicates an esodrift.

Relationships between postoperative stereoacuity measured at the beginning of each follow-up period and postoperative drift are plotted in Figure 1. From postoperative month 1 to the final follow-up, only better distance stereoacuity measured at final follow-up was moderately associated with a lesser postoperative drift on near fixation (rs = 0.484, *p* = 0.002). From postoperative month 6 to final follow-up, better distance stereoacuity at the final follow-up was moderately associated with a lesser postoperative drift at both distance (rs = 0.358, *p* = 0.025) and near (rs = 0.379, *p* = 0.017, respectively). No significant correlation was found between the postoperative drift and the sensory fusion status, as well as fusional convergence amplitude.

Figures 1(a) and 1(b) show the relationship between stereoacuity measured at 1 month postoperatively and the postoperative drift from month 1 to month 6 for near and distance fixation, respectively (rs = −0.17, *p* = 0.31; rs = 0.21, *p* = 0.20, respectively).

Figures 1(c) and 1(d) show the relationship between stereoacuity measured at 1 month postoperatively and the postoperative drift from month 1 to final visit for near and distance fixation, respectively (rs = −0.08, *p*=0.65; rs = 0.02, *p*=0.90, respectively).

Figures 1(e) and 1(f) show the relationship between stereoacuity measured at 6 months postoperatively and the postoperative drift from month 6 to final visit for near and distance fixation, respectively (rs = 0.03, *p*=0.85; rs = 0.13, *p*=0.45, respectively).

### 3.4. Additional Analysis

There was no statistically significant (all *p* > 0.055) difference of postoperative drift between the different types of surgical method, gender, and different types of IXT. No statistically significant correlation (all *p* > 0.059) was found between postoperative drift and any of the following preoperative factors: age at surgery, best-corrected visual acuity, and cycloplegic refraction.

## 4. Discussion

Over the centuries, ophthalmologists, optometrists, and neuroscientists have endeavored to explore the relationship between binocular functions and ocular misalignment. Clinical investigations on IXT on these two topics have been conducted: binocular functions serving as objective measures to assess the severity of this disorder [14, 15, 17] and binocular function serving as sensory outcomes following surgery [24, 25]. In this study, we investigated whether postoperative binocular functions could serve as a predictor for the long-term postoperative stability of ocular alignment. If this is the case, then there would be a link between sensory and motor outcomes. Prior to the study, we had hypothesized that better binocular function following surgery would lead to a more stable ocular alignment. Our findings from the study with a mean follow-up period of 37 months contradict this hypothesis.

Few studies have explored the relationship between postoperative binocular function and the stability of postoperative ocular alignment. Kushner and Morton [26] suggested that improvement in binocular function after surgery (within 6 weeks), which had been detected by Bagolini's lenses, seemed to be related to the long-term stability of postoperative alignment in adults with long-standing constant strabismus. According to Birch and her colleagues [27], nil stereoacuity that developed immediately after achieving alignment (within 3 months) increased the risk rate 3.6 times for reoperation in children with infantile esotropia. Notably, the aforementioned binocular functions in the two studies fall into the “with-or-without” pattern. Additionally, the subjects they studied (i.e., the infantile esotropia and the adult with long-standing constant strabismus) would have a lower chance to regain or achieve bifoveal fixation compared with IXT [1].

Based on our data, clinical measures of postoperative binocular function (e.g., the sensory fusion, stereoacuity, and fusional convergence amplitude) cannot predict the long-term stability of postoperative ocular alignment in children with IXT. One possible explanation is that the angle of deviation [28] and stereoacuity [23], as well as control [29], could vary considerably over the day in patients with IXT, even after surgery. Recent prospective studies applied a nonconsecutive retest procedure to confirm the change in the severity of IXT [30, 31]. However, our patients only had one assessment per follow-up. Another potential explanation might be that the clinical methods used here (e.g., Worth's 4-dot test for fusion; TNO for stereopsis) are designed to identify the degree of impairment rather than to measure individuals' ability [20, 32]. Accordingly, the clinical methods are not as accurate as those lab-based methods. For example, Holmes et al. reported that most patients with IXT show normal near stereoacuity [33] detected by preschool Randot's test. However, Wu and his colleagues found that the temporal integration for stereopsis is impaired in IXT patients using a computer-generated random dots paradigm [34]. Therefore, the underlying reason might lie in our inability to detect the impairment of binocular functions with the currently used methods. Hence, a future well-designed prospective study is needed to resolve these issues.

We found moderate associations between distance stereoacuity measured at the final visit and the exodrifts. Though without predictive value, these results indicate that patients who exhibited better distance stereoacuity have experienced lesser exodrift. Our findings, therefore, suggest a plausible hypothesis that might be explored in future studies: larger exodrift might disrupt distance stereoacuity. This hypothesis is not true for the untreated patients, where the Pediatric Eye Disease Investigator Group (PEDIG) found that larger angle of deviation was not associated with poorer stereoacuity [35].

Our data confirm the results of several studies [14, 16, 24, 25]: distance stereoacuity and central fusion improved following surgery in patients with IXT. Although no statistical difference was found, there was a trend that near stereoacuity and peripheral fusion improved after surgery. Furthermore, we found that these sensory improvements did not significantly decline during a mean follow-up of 37 months.

We show that there was a significant postoperative drift toward exodeviation, be it short-term period (from postoperative month 1 to postoperative month 6) or long-term period (from postoperative month 1 to final follow-up); this finding corresponds to the high recurrence rate reported previously in patients with IXT [8–11]. The mean exodrift on distance fixation from postoperative month 1 to final follow-up (37.2 ± 7.9 months) was 7.7 pd, which was much similar to the previous studies. Pukrushpan and Isenberg [36] reported a mean 8.2 pd exodrift for exotropia from week 1 to 36 months postoperatively. Scott and colleagues [21] found an exodrift ranging from 6 to 11 pd for exotropia from postoperative week 1 to 2 years, with the IXT group showing slightly greater amount of drift.

This study, along with previous studies [21, 36, 37], suggests an existence of exodrift in IXT postoperatively. A possible explanation is that the surgery effect regresses over time, which receives support from the adaptive changes of the extraocular muscles [38] and the active neural plasticity [39] after strabismus corrective surgery in the animal model. While, from another aspect, would the progressive exodrift following surgery reflect the natural course of IXT? Up to now, the natural course of IXT has been quite complex and controversial. The conventional view takes IXT as a progressive disorder [1]. In a population-based study, Nusz et al. [40] found that more than half of the patients would increase their exodeviation with 10 or more pd within 20 years of follow-up, and 3.6% of their patients resolved spontaneously during their follow-up. They also reported that patients who had resolved over time tended to have smaller angle of deviation and better stereopsis. However, several studies suggested that IXT had a stable course and even got resolved spontaneously [41, 42]. Recently, PEDIG [30] conducted a randomized clinical trial, which suggested stable exotropia control, stereoacuity, and magnitude of deviation over 3-year observation in children with untreated IXT. According to the data, the baseline stereoacuity seems to be unpredictable of the change of the ocular alignment. Based on 89 patients with a follow-up of 5 years, Kwon et al. [37] found that nearly 90% exodrift occurred in the beginning of the two-year period following surgery. It remains unknown whether the regression effect or the nature course should be responsible for the progressive exodrift following surgery.

In this study, we had chosen postoperative month 1 as baseline to determine the postoperative drift. Our choice enabled us to eliminate potential immediate responses and complications from surgery, such as pain, edema, bleeding, and reactions to anesthetic. Furthermore, the recovery period after surgery allowed patients to restore or develop their binocular function. In addition, we had set relatively strict inclusion criteria to minimize the influence from other factors, i.e., the amblyopia, the anisometropia, and the vertical deviation. For this reason, we excluded patients with overcorrection at 1 month postoperatively, which has been suggested to be correlated with long-term motor benefit but potential threat to binocular function [9, 11, 21]. However, it has been suggested that the fastest and greatest amount of exodrift was observed during postoperative 1-month period, especially for those patients with initial overcorrection at 1 week postoperatively [12, 43, 44]. We, therefore, also collected patients' angle of deviation at 1 week postoperatively to determine the influence from immediate postoperative period. The angle of exodeviation was 0.7 ± 2.3 pd (range: −4∼10 pd) at distance and 0.6 ± 2.2 pd (range: −4∼10 pd) at near. One of the patients had esotropia (4 pd at both distance and near) at 1 week postoperatively. The mean exodrift was 1.4 ± 3.2 at distance and 1.1 ± 3.1 pd at near from postoperative week 1 to month 1. These results indicate that initial overcorrection is less likely to confound the main purpose of this study.

Our study has limitations. First, a selection bias occurred due to the retrospective design of data collection. Secondly, control score [2], which is informative for assessing patients' ability to control exodeviation, is absent in our study and cannot be directly inferred from stereoacuity or angle of deviation [35].

In conclusion, our findings suggest that clinical measures of sensory fusion, fusional convergence amplitude, and stereoacuity cannot serve as a robust predictor for long-term stability of postoperative ocular alignment in patients with IXT who underwent successful surgery without overcorrection at 1 month postoperatively. We hope that our study can motivate more studies that investigate the relationship between binocular function and the stability of ocular alignment, thereby providing new insights into IXT and guiding clinical practice.

## Figures and Tables

**Table 1:** Demographics and clinical characteristics of 39 patients with IXT.

Characteristics	
Gender: female, male	14, 25
Age at surgery: median (quartiles), years	7 (5, 9)
Preoperative deviation at distance: mean ± sd (95% CI), pd	38.8XT ± 8.5 (35.9–41.7)
Preoperative deviation at near: mean ± sd (95% CI), pd	34.4XT ± 10.1 (31.1–37.7)
Exotropia type	
Basic	29 (74.4%)
Convergence insufficiency	10 (25.6%)
Pseudodivergence excess	0 (0%)
True divergence excess	0 (0%)
Surgical method	
BLR	8 (20.5%)
URR	28 (71.8%)
BLR + UR	3 (7.7%)

XT = towards exodeviation; BLR = bilateral lateral rectus recession; URR = unliteral lateral rectus recession and medial rectus resection; BLR + UR = bilateral lateral rectus recession and unliteral medial rectus resection.

**Table 2:** Success rate for different surgical types.

Follow-up		Method	Surgical outcomes		
			Success (%)	Undercorrection (%)	Overcorrection (%)
6 months		BLR (*n* = 8)	6 (75)	2 (25)	0 (0)
		URR (*n* = 28)	21 (75)	7 (25)	0 (0)
		BLR + UR (*n* = 3)	3 (100)	0 (0)	0 (0)

Final visit		BLR (*n* = 8)	4 (50)	4 (50)	0 (0)
		URR (*n* = 28)	15 (53.6)	13 (46.4)	0 (0)
		BLR + UR (*n* = 3)	2 (66.7)	1 (33.3)	0 (0)

BLR = bilateral lateral rectus recession; URR = unliteral lateral rectus recession and medial rectus resection; BLR + UR = bilateral lateral rectus recession and unliteral medial rectus resection.

**Table 3:** Postoperative exodrift between subgroups.

Exodrift∗ (pd)	Distance measurement										Near measurement									
	Fusion					Stereoacuity					Fusion					Stereoacuity				
	Present	*n*	Absent	*n*	*p* ^§^	High-grade	*n*	Moderate-low-grade	*n*	*p* ^§^	Present	*n*	Absent	*n*	*p* ^§^	High-grade	*n*	Moderate-low-grade	*n*	*p* ^§^
From postoperative month 1 to month 6^†^	4.4 ± 6.6	30	1.4 ± 4.9	5	0.45	2.0 ± 7.9	13	4.1 ± 5.6	26	0.20	3.3 ± 5.8	35	—	0	—	3.7 ± 6.2	20	2.7 ± 5.1	19	0.63
From postoperative month 1 to final follow-up^†^	7.3 ± 7.7	30	5.8 ± 9.3	5	0.63	7.5 ± 7.2	13	7.8 ± 10.2	26	0.97	7.2 ± 8.6	35	—	0	—	8.6 ± 8.9	20	7.1 ± 10.1	19	0.57
From postoperative month 6 to final follow-up^‡^	3.6 ± 9.6	31	1.8 ± 4.3	4	0.67	4.8 ± 8.7	15	3.9 ± 11.6	24	0.86	4.9 ± 9.4	37	—	0	—	4.3 ± 7.8	24	5.3 ± 11.9	15	0.68

Patients were divided into 2 subgroups according to (1) Fusion (tested with Worth 4 dots): Present (4 dots) vs Absent (2 or 3 dots) and (2) Stereoacuity: High-grade (≤ 60 arcsec) vs moderate-low-grade (> 60 arcsec). ^∗^Data are presented as mean ± sd; A positive value means a drift towards exodeviation. ‡Patients were divided into subgroups based on binocular function measured at 1 month postoperatively. ‡Patients were divided into subgroups based on binocular function measured at 6 months postoperatively. §two-side Wilcoxon rank-sum test, α = 0.05.

## Data Availability

The data used to support the findings of this study are available from the corresponding author upon request.
